# Technologically-enhanced psychological interventions for older adults: a scoping review

**DOI:** 10.1186/s12877-020-01594-9

**Published:** 2020-06-04

**Authors:** F. Vailati Riboni, B. Comazzi, K. Bercovitz, G. Castelnuovo, E. Molinari, F. Pagnini

**Affiliations:** 1 Department of Psychology, Università Cattolica del Sacro Cuore, Milan, Italy; 2 IRCCS Santa Maria Nascente, Fondazione Don Gnocchi, Milan, Italy; 3 Department of Psychology, Harvard University, Cambridge, MA USA; 4 Istituto Auxologico Italiano IRCCS, Psychology Research Laboratory, Piancavallo, Verbania Italy

**Keywords:** Older adults, Aging, Technology, Psychological interventions, Clinical psychology, Healthcare

## Abstract

**Background:**

The world population is getting older. As life expectancy increases, traditional health care systems are facing different challenges in terms of cost reduction and high-quality service delivery capability. New ways to improve older adults’ quality of life have been explored, taking advantage of new technological solutions. Our focus is on the integration of technology in clinical treatments to facilitate or deliver psychological interventions meant to improve well-being in older adults. Our aims were to describe the main technology-based interventions supporting seniors’ quality of life or psychological well-being and to provide greater clarity to what is described in the current literature as their effects on seniors’ cognitive and psychological outcomes and healthcare policies.

**Methods:**

We reviewed the scientific literature looking for studies that investigated how technology can be implemented into clinical psychology treatments for older adults. Our search was conducted using the following databases: *PubMed*, *PsycINFO*, *Scopus*, *ISI Web of Science*, and *CINAHL*. The search provided 350 articles, mostly (≈90%) dated after 2002. Abstract analysis narrowed the selection to 150 papers, according to their relevance and actuality as judged by a restricted group of independent researchers.

**Results:**

Through a thematic analysis, we found that virtual reality (VR), robots, telemedicine, software, video games, and smartphone applications could potentially support older adults’ psychological treatment with a positive impact on healthcare systems.

**Conclusion:**

Findings from the literature are encouraging, although most of these results are only preliminary.

## Background

The world population is getting older, as life expectancy increases and the birthrate is lower than in previous decades. In the European Union, the percentage of people 65 and older will increase from 25.4% in 2008 to a predicted 53.5% by 2060 [1, 2]. Worldwide, the population over 60 years old is currently about 900 million and expected to reach two billion by 2050 [1]. Further relevant data comes from analyses conducted by the Centers for Medical and Medicaid Services, highlighting that 63% of the older adults have been diagnosed with a chronic condition [2]. Modern healthcare systems appear to be failing in the treating of chronic symptoms, with negative long-term economic consequences [2]. Demographic changes require new strategies and new developments in terms of research dealing with well-being issues and quality of life maintenance in later life. As these changes in age seem to indicate, the problem of ‘adding years to life’ may become secondary to that of ‘adding life to years’ [3]. The psychological empowerment of healthy older adults has received increased emphasis in the last decade [1, 4]. Psychological interventions have been recognized as being both clinically successful and cost-effective in the promotion of seniors’ well-being and mental health [5–7]. What appears to be still partially unexplored, however, is the potential of integrating new technologies into psychological initiatives for individuals in their later life. Advanced technology innovation within the framework of senior care could be a possible solution to the negative effects of the worldwide aging process that actively addresses the challenges of an aging population [8, 9]. Many technological advances are dealing with health and social outcomes [10, 11]. Technologies can support well-being in older adults in different ways. For example, encouraging different lifestyles [10], providing non-invasive assessments [11], and delivering distance interventions [12]. Technology that meets the needs of older adults is labeled Gerontechnology, aimed at supporting successful aging in a way that encompasses the full range of human activities [12, 13].

Exploring and expending the suitable forms of technology-enhanced psychological interventions in older adults health care appears to be a promising strategy, given its potential to reach a high percentage of the population, to decrease the number of personnel required to treat each patient, and to improve accessibility to efficient health care services [14, 15].

The focus of the current review is the exploration of how new technologies have been used to enhance psychological interventions with older adults, to promote improved quality of life and psychological well-being. To assess the state of the art on technologically-enhanced psychological interventions for older adults, our scoping literature review will be addressing the two following research questions:

1. What are the main technology-based interventions supporting seniors’ quality of life or psychological well-being?
2. What are their effects on cognitive and psychological outcomes, and what is their potential economic impact?

## Methods

The current study has been developed as a literature scoping review, an increasingly adopted approach for reviewing evidence from health-related research [16]. Although the literature lacks one complete agreement on the definition or purpose of scoping reviews, most explanations addressed it as a research process with the specific objective of summarizing evidence to communicate “the breadth and depth of a field” [16]. Scoping reviews do not rigorously weigh studies quality, like systematic reviews [17]. Moreover, scoping reviews adopt broader research questions or inclusion/exclusion criteria, may not request extraction of the data and usually present a higher qualitative VS quantitative results ‘discussion [16–18].

We screened the scientific literature, searching for papers that included the use of some form of technology to improve or to deliver a psychological intervention, with a specific focus on the aging population (65+). Different kinds of experimental studies were considered, including RCTs, longitudinal designs and qualitative research. Reviews were also included as a source of aggregated information. Our search was conducted through the following databases: *PubMed*, *PsycINFO*, *Scopus*, *ISI Web of Science*, and *CINAHL*. The searches included the following terms: (“older adults” OR “ageing” OR “aging” OR “elderly”) AND (“psychological interventions” OR “clinical psychology” OR “psychotherapy” OR “counseling”) AND (“technology” OR “Tech device” OR “Gerontechnology” OR “e-Health”). These terms were searched as keywords, titles, abstracts, and MeSH. Additionally, citation maps were examined and the ‘cited by’ search tools were used where available. Unpublished works were not considered. Study selection was guided by the researcher’s inclusion criterion for articles in which technology, in its various forms, was the only and exclusively used intervention on the older adult population (i.e. no additive face-to-face support).

Another inclusion criterion was the presence of specific psychological (i.e. well-being, anxiety, depression, loneliness) and cognitive outcomes (i.e. memory, attention, processing abilities), both in terms of prevention and health promotion targeted by technological intervention.

A further inclusion criterion was the appearance of references to real economic or potential savings factors for the healthcare system.

One more fundamental aspect was the replicability of the studies outside of an exclusively experimental context. All selected papers met the inclusion criteria, except for the economic-related criterium, which was considered an optional factor to better review the economic impact of the different interventions examined in the scoping review.

**Fig. 1:**
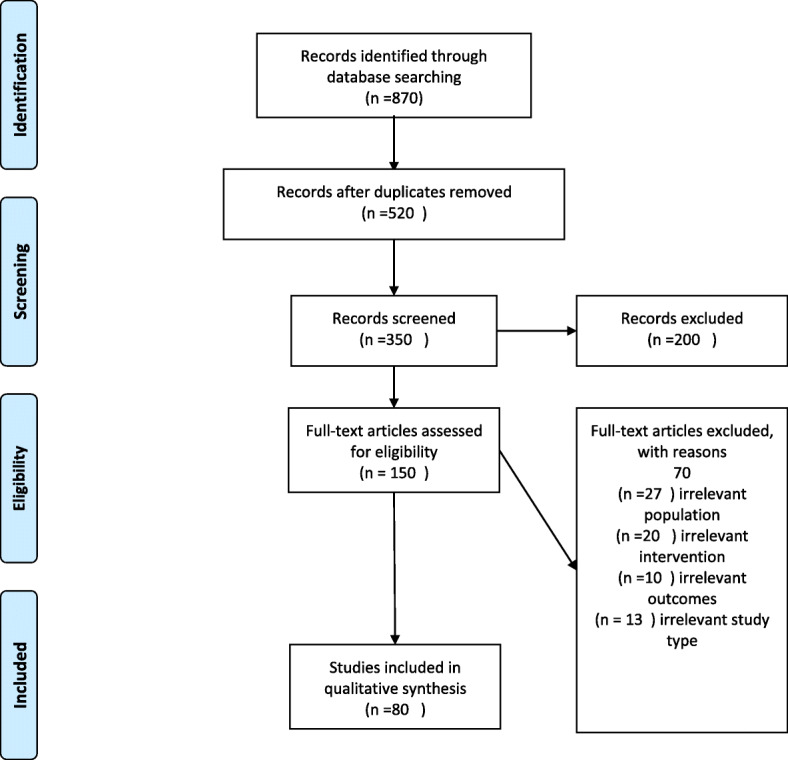
PRISMA 2009 Flow Diagram

PRISMA guidelines were followed, and the flow chart is shown in Fig. 1. The first and second authors collaborated in the articles screening process, independently. When discrepancies emerged, the last author was consulted to obtain complete agreement. A total of 870 records was identified through the database searching using the keyword search listed above. After duplicates identification and removal, both abstracts and titles were analyzed, excluding irrelevant studies in line with the prior specified inclusion criteria.

Hundred and fifty full articles were then read entirely by the first, second and last author, and records were then cut out in the presence of any of the following exclusion criteria: irrelevant targeted population (population younger than 65 years old), irrelevant technological intervention (not exclusively technology-based intervention), irrelevant outcomes (absence of psychological or cognitive outcomes directly targeted by the intervention), or irrelevant study type (studies focused on the description of technologies without a clear intervention on older adults).

Eighty articles were identified as appropriate for inclusion in the present review. Data extracted were: study’s purpose, design, and methodology of the study, targeted population, form of technology reported, measured psychological and cognitive outcomes. When data extraction was completed, a sub-sample of thirty papers was reviewed by the last author, ensuring data characterization’s validity. In line with Arskey and O’Malley guidelines, a thematic content analysis approach was carried out to identify common and distinctive themes from each form of technology reported to improve or to deliver psychological interventions to older adults, according to inclusion criteria [17].

Discovered themes were organized, discussed and theoretically classified by the authors to facilitate comparisons.

Accepted studies were classified by the different forms of the technology described. To avoid the risk of bias, categories were not predefined by the authors and the classification was entirely conducted retrospectively.

## Results

Eighty articles were considered for this review. Studies were labeled according to the characteristic of the different technology-based interventions supporting seniors’ quality of life or psychological well-being.

Through a thematic analysis, we classified the papers into six categories based on the technology addressed:

1. Virtual reality (VR),
2. Robots,
3. Telemedicine,
4. Smartphone Apps,
5. Software,
6. Videogames.

Within each category, definitions and practical examples are presented, describing the typical features of each approach, its strengths and its clinical value. A summary of their effects on older adults ‘cognitive and psychological outcomes, as well as on healthcare policies will be addressed for each category.

### Technological solutions to implement psychological interventions

#### Virtual reality

Virtual reality (VR) refers to an artificial environment, created with software, that resembles a ‘real’ environment in some way [19]. It is generally experienced through sight and sound with the help of a computer. Although research on this construct has been produced for decades, the use of virtual reality for the implementation of psychological interventions has become very popular in recent years, with a significant increase in the quality of the studies and systems used [20–22]. Previous studies, conducted with a general population, have suggested that a VR-implemented psychological protocol can be efficacious in treating a variety of psychological disorders and behavioral issues [21, 23, 24]. Available findings report benefits, after applying psychological interventions involving VR, in terms of prevention of age-related cognitive decline. For example, one study found that older adults who underwent intense six-month VR memory training, involving auditory stimulation and VR experiences in pathfinding, showed improvements in memory tests, especially in the long-term recall, in comparison with those in a control group who demonstrated a decline [25]. Moreover, virtual reality training may exert a positive effect on motor balance. A research protocol has been proposed to test the hypothesis that treadmill training using virtual reality may improve gait and balance in older adults, people with mild cognitive impairments and people with Parkinson’s disease [26]. The study is currently ongoing.

The application of VR to psychological interventions for the promotion of well-being in older adults seems promising and feasible, offering significant advantages over conventional treatments in terms of functionality, subject accessibility to a wider number of test or clinical stimuli, subject stimuli interaction, standardization of experimental treatments, treatments’ environmental manipulation possibilities and subjects’ safety conditions, although there is a need for a more controlled longitudinal study exploring the hypothesis [27]. VR could be useful in reducing anxiety when coupled with cognitive-behavioral therapy (CBT), which uses traditional exposure techniques, because exposure therapy tends to be very effective in younger populations, but the creation of vivid/detailed mental images is sometimes impaired in older adults, preventing successful treatment [28, 29]. VR environments can supplement the lack of vivid images and memories, allowing the anxiety stimulus to be fully introduced in the therapeutic process [27]. One common limitation of the VR intervention shared by the studies considered is the results ‘transferability to real-world abilities or daily living activities. Older adults’ improvements in the experimentally targeted outcomes are usually assessed with instruments that fail to allow a generalization of the results on a wider pattern of situation.

A further limitation commonly described in the literature deals with the side effects due to head-mounted VR devices [27]. Although this appears not to be specific to the older adult population, many studies have underlined different issues such as nausea, headache or disorientation in VR users. Finally, one more limitation often mentioned in the studies considered is related to the economic cost of VR [29, 30]. However, technological progress has already allowed the creation of VR devices accessible to a higher percentage of the population. Unlike years ago, a full VR equipment (one computer, one head-mounted display, and one motion sensing input sets) can be bought for less than $1500.

Concerning VR acceptance in clinical practice by psychotherapeutic staff, the literature suggests encouraging results, with data indicating that for an increasing percentage of therapists, obtainable outcomes through VR devices overcome the possible costs of the devices themselves [30].

#### Robots

The rapid technological advancement of the last decades has produced robots not only for industrial production but also for dynamic interactions with humans [31]. Robots for psychological enrichment, designed to entertain, communicate, educate and rehabilitate, have been developed and tested. Broekens (2009) proposes a double categorization of the robots most used today: rehabilitation robots and assistive social robots [31]. Those of the first kind are usually not communicative and concentrate on the physical rehabilitation of the subject, while the second kind can support patients in basic duty promoting and increasing the level of independent living ability or those aiming to increase the patient’s level of psychological wellbeing. Shibata and Wada (2011) compare the development of this technology, which is quite common in Japanese hospitals, to widely-used pet therapy [32]. However, the presence of animals in elder care settings involves many risks: they could bite, may not adequately respect hygienic standards and require great attention, affecting both time and organizational resources [33]. Research presenting companion robots’ advantages for patients, in terms of brain function and stress hormone production, is encouraging [34–36]. Most of these robots are designed to stimulate a positive emotional reaction in people. For that reason, various types of shapes have been developed, including humanoids, animals and other structures [32]. From a methodological point of view, however, it is not possible to distinguish which of these shapes could potentially bring the most significant effect on the patient. In the studies considered, critical issues are limiting internal and external validity [31]. The first thing to consider is that most of the experimental research in this field mainly uses a single form of robot, a seal-like called Paro, in the absence of comparison with other shapes [31]. Moreover, most the experiments take place in Japan, within nursing homes, through experimental designs that are not able to significantly control possible intervening variables and outcomes that are difficult to interpret [31].

Interactive autonomous robots connect with people by using verbal and non-verbal communications [31, 37]. They can process information and respond to stimuli with different levels of complexity. The robot most frequently used in this way is Paro, a seal-like robot designed to stimulate feelings such as pleasure and relaxation [38]. The responses that it provides when it interacts with people can be interpreted, on a behavioral level, as if the robot has feelings. On this basis, Paro was used as an example of ‘robot therapy’, with a particular focus on the care of younger children and older adults [35]. The presence of this robot at a day service center for older adults promoted a reduction in stress and depressive symptoms after 5 weeks [39]. Moreover, similar results were obtained from long-term interaction, and caregivers reported that interaction with the robot helped older people to become more active and smile more [40, 41]. Concerning some of the effects presented in the Paro-related literature, it must be highlighted that most positive outcomes seem to be significantly connected to the older adult’s baseline health condition. Such as that better results can be expected in less severely affected participants [42].

A few studies have shown further benefits, providing encouraging results in contrast to the hypothesis that robotic devices only focus on an emotional level, but can be used significantly as cognitive stimulators, optimizing both seniors’ interactivity and processing ability [35, 43]. Results from robotic psychological interventions are usually described in terms of better or improved older adults related outcomes, like more efficient cognitive or neural functioning, improved anxiety coping abilities, or better quality of life [44].

However, one limitation of some studies included in this scoping review, especially those dealing with older adults with dementia or mild cognitive impairment is that those observed positive outcomes often imply less decline, rather than real positive improvements [44–46].

One more limitation of the literature on robotic psychological interventions is the paucity of a randomized controlled trial comparing the effects of specific robots’ enhanced treatments with others involving regular psychological interventions [46–52]. More importantly, of those few published studies, some showed conflicting results from the robot condition, with specific assessed outcomes measures appearing inferior [45, 51]. In one study, the negative effects of the robot’s treatment on the older adult population, such as increased irritability or lability within the symptoms have been described [53]. Whether or not robots enhanced psychological interventions could be triggering anxiety or negative emotions in older adults need further and more controlled investigations.

The use of robots in mental healthcare for older adults is an emerging field, underlying the crucial role those kinds of machines could have on different health care policy factors, such as experience, finance, capacity and quality [54]. Exploratory experiments suggest that there is a potential, at least in terms of acceptability and feasibility [55]. Robotic diffusion in clinical practice, however, still seems to be slowed down by the cost of production, although more and more specialized industries are working on affordably-priced commercial robots. While this area is in constant development, it is open to new studies and new possible applications.

#### Telemedicine

Telemedicine is usually defined as the exchange of medical information from one site to another using electronic communications, aimed at improving patients’ clinical health status [14]. It involves the use of technology to deliver care to a person that is remotely located from the provider; it can also assist with clinical decision-making [56]. There are two main delivery systems for telemedicine, one through the phone line and the other one via the internet (e.g., video chats, emails, text chats). Most extant literature about telemedicine and older adults investigates the use of medical devices that constantly monitor the person’s biological data, focusing especially on people with cardiovascular diseases and diabetes [57–59]. Some of these monitoring activities have been tested with people with dementia [60], while other forms of distance support have been explored with their caregivers, aimed at reducing their burden [61]. As observed by Boaz and colleagues, telemedicine interventions targeting biological outcomes may not be more efficient than other regular treatments [56]. However, a positive impact on psychological variables such as well-being or quality of life could be more easily achieved. The remote monitoring of the patient’s care and health through telemedicine interventions could, offer a positive strategy to increase older adults’ sense of control over their health conditions. Thus potentially showing positive repercussions on the psychological well-being of the older individual [62, 63].

Seniors’ attitude can be a hindrance to the acceptance and use of new technologies with which they are not familiar [64]. It seems that positive and negative attitudes toward technology could be related to an active or passive role, respectively, that people have in the learning process [64]. Despite potential benefits in terms of assistance and independence, the current literature emphasizes some limitations in the application of telemedicine for older adults [57]. That could be the reason for the paucity of experimental longitudinal research, especially when compared with the number of feasibility and pilot studies that are carried forward over time, becoming widely used and adopted systems [65, 66].

Consumers’ perception of benefits has been described as one of the most influential barriers to acceptance within the senior population, while convenience and daily activity integration possibilities identified as other mediating variables [67]. As Jimison suggests, strategies aimed at increasing older people’s active engagement with telemedicine could lead to significant positive treatment outcomes and high acceptance levels [67]. Telemedicine is considered to have great benefits for both primary and secondary care [68]. Experiments conducted on older people have shown promising results. Spek and colleagues [69] reported that an internet-based cognitive behavioral self-help intervention could decrease depression in a sub-clinical population of older adults, with results that persisted for over a year [70]. Titov and colleagues examined the efficacy of an internet-based cognitive-behavioral therapy (CBT) program for older adults with anxiety [61] or depression [71], which is structured over 8 weeks and includes online lessons, homework activities and distant interaction (email or phone calls) with a therapist. In both cases, the program demonstrated good efficacy in symptom reduction compared to a waitlist control group. The treatment was also effective in terms of costs and impact on their quality-adjusted life years [71, 72]. Some studies have intensively analyzed the benefits of adopting telemedicine in terms of clinical results and cost-efficacy. For example, the Veterans Health Administration (VHA) has achieved important results introducing a telemedicine program for their patients. They were able to reach up to 119,535 subjects, generating savings of about $1999 per patient per year. At a clinical level, 36% of the veterans treated with telemedicine were able to increase their independent living level [12]. Hospitalizations decreased by 38% and in-care treatment decreased by 58% [12]. In another telemedicine program, conducted at Partners Health Care, 3000 subjects were treated with in-home monitoring technology, so that factors such as blood pressure, heart rate, and weight were always under daily direct control. The data collected highlighted a 44% reduction in hospital readmission, generating a cost reduction of up to $10 million in 6 years [73]. As these studies suggest, these positive outcomes could be more easily achieved when specific elements overcoming possible arising senior population barriers are considered. Direct and easily accessible feedback on the patient’s status or a personalized data interpretation based on the patient’s treatment goals and its treatment’s adjustments overtime should always be guaranteed [67]. Another form of telerehabilitation can be seen within the large field of smart home research. Smart home systems enable individual possibilities of living in their own chosen environment, preventing them from institutionalization or nursing home assignment [74].

Sensors can be applied and located in different places in people’s apartments or houses, managing and monitoring risky situations. Different kinds of sensors, such as smoke detectors, flood detectors, motion sensors, automatic controllers, alarms or visitor identification systems are already being tested with promising results [75]. Software services are also a component of the smart home environment. The benefits of this tech application can be synthesized in the promotion of healthier lifestyles for seniors, monitoring caloric intake, vital signs or sleep patterns. The information gained from the smart home environment can help seniors to monitor basic tasks (such as getting dressed and walking around) and instrumental activities (such as telephone calls and web use) in their daily lives while capturing deviations from regular patterns of physical or cognitive well-being [74–76]. By allowing older people to stay in their own houses longer, smart home technology can potentially represent substantial savings for the healthcare system [77].

One study from the University of Missouri calculated how by introducing smart home technologies in seniors’ houses, individuals could benefit from more time in their own place, delaying the need for retirement homes with economic savings to Medicaid of nearly $87,000 [75]. A fundamental limitation factor of smart home technologies can be seen in the technology readiness of the older adult population. Although individuals in their later life are now more engaged in technology innovation, some studies published and considered for this scoping review have been conducted in artificial environments, making generalizations of the results still not clinically valid [78].

#### Software, video games, and smartphone apps

Together with internet-based programs that can deliver a psychological intervention, other technologies can support psychological treatment using a personal computer, such as software, video games, and smartphone applications. According to Kueider, a theoretical classification could be made regarding the type of computerized software used: classic cognitive tasks, neuropsychological software and video games [79]. Significant evidence from the literature suggests that these kinds of technology interventions, aimed at improving older adults’ well-being, can enhance their cognitive performance, with significant outcomes’ effects up to 5 years post-intervention [80, 81]. Despite common age-related stereotypes connected with seniors facing and handling new technologies, most older adults, after reporting increasing anxiety levels at the beginning of the experimental training, showed significantly increased satisfaction and a higher internal locus of control [82]. To ensure a positive experience of these technologies by older users, a possible solution could be achieved by demystifying stereotypes and prejudices regarding aging and seniors’ technology skills. At the same time, special attention must be paid when exposing older adults to new technologies, with professionals trained to improve older subjects’ experience while at the same time providing senior-friendly guidelines [82–84].

Several programs have been developed, although few have been systematically tested using a scientific approach [85, 86]. One of these is the Butler system, developed and tested by a Spanish research team [87]. This is a technological platform that provides three main applications for older adults: diagnosis (mood monitoring, alert system, management reports), therapy (training in inducing positive moods, memory work), and entertainment (email, chat, video, photo albums, music, friend forums, accessibility to the internet). This program can serve as a base for the delivery of different interventions. An example is the implementation of a life-review therapy. Preschl and colleagues [88] explored the effects of a six-week life-review therapy protocol in a face-to-face setting with additional computer use, provided by the Butler system. Depressive symptoms decreased significantly over time in the intervention group compared to the waitlist control group, suggesting the strong potential for this technology’s application. There is also a growing literature about the use of video games with older adults. The applications of these games are mainly on a cognitive stimulation level [79, 89]. A recent meta-analysis [90] suggested that video game training may moderately enhance the cognitive functioning of healthy older adults, producing positive effects on memory, attention, reaction time and global cognition. To maintain these benefits, it also seems important to continue using the games, as the effects of the training tend to decrease when not followed by other sessions [91]. Some of the advantages of using video games to improve older adults’ cognitive functioning are that they are not expensive and that they can be gratifying and fun [92]. A trial involving the use of a real-time strategy game has shown that older people have experienced an increase in executive function and switching between task sets ability. It is of great importance to emphasize that there may be, therefore, a strong correlation between this kind of treatment and improvements in the executive functions, since these functions are linked to a well-functioning frontal lobe, greatly influenced by the ageing process [93]. Smartphone apps are changing the way health can be promoted in every part of society, including in the older adult population [94, 95]. Possible applications include acquiring health information, personal disease prevention and healthy living, self-diagnosis using built-in sensors, and medication compliance promotion [96]. There are mental health apps, such as stress management or relaxation apps, which have the potential to be effective and may significantly improve treatment accessibility [97, 98]. This, however, is a brand new research field, which lacks solid evidence [96]. Most of the studies in the literature seem to highlight a wild range of positive results, this could, however, be explained by publication bias in favor of those papers that include significant positive outcomes. One of the studies included in the present scoping review has not shown any positive or significant statistical improvements in the psychological factors targeted with a mindful based smartphone application for older adults [97]. In our opinion, a limitation of many studies could be represented by the kind of older adult sample used. Factors like the level of education, attitudes towards technology or previous knowledge and skills with apps or smart devices are rarely considered within the analysis measuring treatment’s effectiveness [99]. Unexplored social or personal variables could prove to be important mediators in this kind of technological intervention.

## Discussion

Considering the increasing rate of chronic disease and related issues within the senior population, the need for alternative or improved care and welfare solutions is crucial. While some new studies are exploring the effects of a return to the past [100], the exploration of how current and future technologies can promote healthy aging is relentant. In particular, Gerontechnology and e-health interventions for the elderly represent a promising approach for mental health promotion [101] and potentially helpful instruments for the delivery of psychological interventions. Examples and research listed in the present article are not meant to fully cover the field of ‘psychotechnology’ applications, but rather to provide a broader vision on how technology tolls are considered by healthcare clinicians and policymakers, given their encouraging outcomes extending psychological treatment to a larger population, improving the quality of care while reducing both individual and national expenditure and significantly correlating with important variables such as patient and provider satisfaction [14]. Developing and adapting care systems to digital health technologies could potentially improve convenience as well as research outcomes [102].

Health innovation is still a long-term process. Today’s care systems appear unequipped to deal with the mismatch between demand for and supply of health care providers. Technologically-enhanced psychological interventions can support the creation of a modern and suitable model of healthcare, improving accessibility and quality while decreasing costs. These hoped-for changes could also increase the effectiveness of the treatments already available while finally putting patients in a central and active role by enabling them to participate directly in their own care [14, 103]. With the possible exception of telemedicine and VR, however, further research in this field is warranted. Most of the experimental contributions do not compare the intervention with an active treatment group. Studies that investigate the effects of different media for the delivery of psychological interventions exist in telemedicine [66], but they are rather uncommon when considering other technologies. There are cultural and social variables that are experimentally underestimated [10], which may challenge the internal and external validity of most studies. Seniors’ learning capability with specific technologies appears to be so influenced by cultural variables, such as level of education, previous work experience, and socioeconomic status. In our opinion, integrating more sub-group analysis in technology research ‘protocols could add greater clarity regarding the role of participants characteristic on the potentially assessed outcomes. This simple strategy could help to address the issue of the role of those variables on technology interventions and acceptance by older adults. Cross-cultural studies and research that investigates the role of these mediators are also needed for a significant generalization of the current results.

The main theoretical models used when considering seniors’ technology acceptance or barriers, the Technology Acceptance Model (TAM) and the Unified Theory of Acceptance and Use of Technology (UTAUT), have recently been criticized by researchers as they appeared to be missing significant community-dwelling predictor variables [104]. In line with Peek suggestions, we believe that a possible solution could be achieved by including six specific themes: independent living related challenges, behavioral options, individual thoughts on tech-usability, individual social network influences, organizational influences and the role of the physical environment [104]. Moreover, a possible significant strategy for integrating technology in the daily lives of older people could be to act directly on age-related stereotypes, as numerous studies have shown that the role played by stereotypes is of fundamental importance. Although this appears to be a difficult solution, multidisciplinary approaches that directly involve health policies could offer significant results [105–108].

Evidence from the present scoping review offers greater insights on the best practice or barriers of the different technology adoption. A first factor to be considered should be the proper choice of assessment tools to be used in any technological interventions. Given the difficult process of results generalization to real-world or daily living activities, researchers should move from an efficacy oriented focus towards a more routine-care effectiveness one. Efforts to increase studies ecological validity could provide more clinical relevance and addressed this first limit.

A second relevant key factor emerging from the papers reviewed concerns those side-effects potentially related to the different technologies. Researchers interested in integrating new technologies in their psychological initiatives should, therefore, pay close attention to the older individual preparation before attempting any treatment. Strategies to empower older adults’ readiness with the new technological tools should be implemented before moving to any treatments. As most of the studies in this review seem to indicate, older adults’ acquisition of the different technology-related skills plays a significant role in terms of intervention ‘efficacy.

Overall, evidence for technological-enhanced psychological treatments remains at an early stage. Trials ‘methodological quality is still not sufficient, with regards to sample size, randomizations, presence of follow-up and statistical approach used. Over the next years, many of the current limitations described in the current paper are likely to be addressed. A more accurate perspective on the true potential of those different technology tolls in the psychological promotion of older adults’ well-being is hopefully soon to be reached.

The application of advanced technology to psychological and behavioral programs for older adults has been recognized as a promising solution for many issues, including depression, anxiety and mild cognitive impairment [10, 101, 109]. We believe that the application of technological devices in psychological treatment could be considered by clinicians and researchers in their activities according to their level of experience and the specific need of patience. Research in this field, however, is still in its infancy [109].

## Conclusion

We presented a scoping review of the different forms of technology adopted to improve or to deliver a psychological intervention to older adults. We qualitatively assessed existing good practices, through concrete examples collected in the literature, and outlined how technology adoption could deal with cost-related problems arising in the health care system. Six broad categories of technology have been identified in this study. Findings suggest how different technology could be used to assess a variety of older adults ‘conditions, with promising results. Given the scoping nature of this study, future systematic or meta-analytic works might concentrate on the topic, to empirically compare the effectiveness of those forms of technology in health care and health-related policy.

## Data Availability

All data and material are listed in the article.

## Abbreviations

NIA: National Institute on Aging-Alzheimer’s Association
CBT: Cognitive behavioral therapy
VR: Virtual reality
TAM: Technology Acceptance Model
UTAUT: Unified Theory of Acceptance and Use of Technology
VHA: Veterans’ Health Administration
